# Concordance of PD-L1 expression and CD8^+^ TIL intensity between NSCLC and synchronous brain metastases

**DOI:** 10.17305/bjbms.2019.4474

**Published:** 2020-08

**Authors:** Sebnem Batur, Onur Dulger, Sermin Durak, Perran Fulden Yumuk, Hale Basak Caglar, Emine Bozkurtlar, Suheyla Bozkurt, Ebru Tastekin, Irfan Cicin, Rengin Ahiskali, Rashad Rzazade, Asli Cakir, Buge Oz

**Affiliations:** 1 Department of Pathology, Cerrahpasa Medical Faculty, Istanbul University-Cerrahpasa, Istanbul, Turkey; 2 Department of Internal Medicine, Division of Medical Oncology, Pendik Education and Research Hospital, School of Medicine, Marmara University, Istanbul, Turkey; 3 Department of Radiation Oncology, Medipol University, Istanbul, Turkey; 4 Department of Pathology, School of Medicine, Marmara University, Istanbul, Turkey; 5 Department of Pathology, Medical Faculty, Trakya University, Edirne, Turkey; 6 Department of Internal Medicine, Division of Medical Oncology, Trakya University, Edirne, Turkey; 7 Department of Radiation Oncology, Anatolia Health Center, Gebze, Turkey; 8 Department of Internal Medicine, Division of Medical Oncology, Medipol University, Istanbul, Turkey

**Keywords:** Programmed death-ligand 1, non-small-cell lung cancer, NSCLC, tumor immunology, PD-L1, brain metastasis, CD8 lymphocytes, tumor-infiltrating lymphocytes, TILs

## Abstract

Programmed death-ligand 1 (PD-L1) is suggested to be a predictive biomarker in non-small-cell lung carcinoma (NSCLC). However, the differential expression of PD-L1 in primary lung tumor vs. synchronous metastases, especially brain metastasis (BM), remains unclear. This study assessed the concordance of PD-L1 expression on tumor cells and tumor-infiltrating lymphocytes (TILs) and CD8^+^ TIL intensity between primary lung tumors and synchronous BMs from 24 NSCLC patients. PD-L1, CD3, and CD8 positivity was determined by immunohistochemistry (IHC). PD-L1 scoring was based on the proportion of tumor cells with membranous expression of PD-L1 and the cutoff values <1%, 1–49%, and ≥50%. CD3 and CD8 positivity in TILs was evaluated semi-quantitatively and the proportion of CD3^+^/CD8^+^ TILs was determined. PD-L1 expression on tumor cells and TILs was evaluated in relation to CD3^+^/CD8^+^ TIL proportions and the intensity of CD8^+^ TILs between the paired primary lung and BM tissues. In the primary lung tumors, PD-L1 positivity was observed in 25%, 37.5%, and 37.5% cases for the cutoff values <1%, 1–49%, and ≥50%, respectively. PD-L1 expression on tumor cells was strongly correlated between the paired primary lung and BM tissues, in all cutoff groups. However, PD-L1 expression on TILs and the proportion of CD3^+^/CD8^+^ TILs were not strongly correlated in all three groups between the paired primary lung tumors and BMs. The intensity of CD8^+^ TILs was concordant in only 54.16% of the paired primary lung tumors and BMs. This study showed a high concordance of PD-L1 expression in neoplastic cells between primary NSCLC and synchronous BMs.

## INTRODUCTION

Immunotherapies targeting the programmed cell death protein 1 (PD-1) and/or programmed death-ligand 1 (PD-L1) are relatively new and promising cancer therapeutic options. Since the discovery of PD-1/PD-L1 pathway and its role in the immune escape of tumor cells, many studies have been investigating the mechanism of how tumor cells block the immune system and progress using that and similar receptor signaling pathways. PD-L1 and PD-L2 are members of the B7 family. They are inhibitory cell-surface protein ligands that inhibit T cells by binding to the PD-1 receptors and can induce apoptosis. Under normal conditions, this is an important step in the immune response that prevents tissue damage caused by induced inflammation. However, in cancer, PD-L1 and PD-L2 protect cells from direct attack by cytotoxic T cells and provide an escape from the host immune system. Such tumor microenvironment facilitates the proliferation of tumor cells. Many studies have shown successful outcomes of PD-1 and PD-L1 targeting immunotherapies in non-small cell lung carcinomas (NSCLC) [1-7]. Moreover, Liu et al. reported an increased survival rate in glioblastoma multiforme patients with upregulated PD-L1 expression in neurons adjacent to tumors [8].

Tumor-infiltrating lymphocytes (TILs), especially T cells, are a part of the tumor microenvironment. Some studies suggested that the CD8^+^/CD4^+^ TIL ratio may predict response to anti-PD-1 treatment in metastatic NSCLC [9-11].

The expression of PD-L1 in tumor microenvironment is dynamic and shows intratumoral heterogeneity [12]. PD-L1 is used as an immune-related biomarker and PD-L1 score gives an estimation of PD-L1 presence in tumor tissue.

Brain metastasis (BM) is very common in NSCLC patients. In terms of metastasis, however, the brain is different from other organs due to its unique microenvironment. This necessitates a different therapeutic approach to BMs. In many cases, the symptoms of lung cancer emerge due to BM instead of primary lung tumor. Moreover, in some cases, even smaller brain lesions of 3–5 mm can cause symptoms, while lung tumors of 6 cm do not. Brain lesions are more life-threatening than lung lesions, as they may cause malfunction of the vital areas of the brain and suppress various metabolic functions [13,14].

Only a few studies have investigated the differential expression of PD-L1 in primary lung tumor vs. synchronous metastases, especially BM [9,15]. In the case of BM in NSCLC, complete or partial surgical removal of metastatic lesions from the brain is much more common than the removal of the primary tumor from the lungs. If tumor has spread to a distant organ, usually there are also lesions in various lymph nodes, and the removal of the primary tumor is not considered to be an effective treatment in such cases [16]. This creates a precarious situation where the lesions in the brain may be removed by surgery, while the primary lesion in the lung is not operated. Even when stereotactic radiosurgery is an option, some tumors are just too large to be effectively managed by radiotherapy, and classical surgery remains as the only option. This is important because, in many cases, the tissue removed from the brain is used as a biopsy material for various pathological analyses, including the determination of PD-1 and PD-L1 expression. In this sense, determining the concordance in PD-1 and/or PD-L1 expression between primary lung tumor and BM is important to be able to accurately determine the suitability of patient for immunotherapy [17].

This study assessed the concordance of PD-L1 expression on tumor cells and TILs and CD8^+^ TIL intensity between primary lung tumors and synchronous BMs from 24 NSCLC patients.

## MATERIALS AND METHODS

### Patients

Twenty-four patients diagnosed with primary NSCLC and synchronous brain metastases were selected (13 cases from Cerrahpasa Medical Faculty, 6 cases from Marmara University Medical Faculty, 3 cases from Medipol University, and 2 cases from Trakya University Medical Faculty). BM tissue specimens were removed before neoadjuvant therapy. This allowed the comparison of PD-L1 expression and TILs between primary lung tumors and their synchronous BMs without the influence of therapy. Resected specimens of BMs and resected and biopsy specimens of primary lung tumors were stored as formalin-fixed paraffin-embedded (FFPE) tissue samples.

A total of 48 FFPE tissue blocks were sectioned at 5 µm. The palatine tonsil was used as the control tissue.

### Immunohistochemistry (IHC)

PD-L1, CD3, and CD8 positivity was determined by IHC. CD3 and CD8 IHC was performed on a BenchMark ULTRA Autostainer (Ventana Medical Systems, Tucson, AZ, USA). CD3 positivity was evaluated semi-quantitatively (minimal, moderate, or severe) and CD8 positivity was scored on a percentage scale over CD3 positive cells.

IHC for PD-L1 was carried out using a DAKO Autostainer Link 48 and monoclonal mouse anti-PD-L1 clone 22C3 PharmDx (Dako, Carpinteria, CA). In tumor cells, positive PD-L1 staining was defined as either partial or complete membranous staining at any intensity. Necrotic areas were excluded from the scoring. The percentage of PD-L1(+) tumor cells was determined and the samples were considered ‘positive’ if ≥1% of the tumor cells were stained. We used three cut-off values for PD-L1 staining of tumor cells: negative (<1%), poor positive (1–49%), and strong positive (≥50%). In TILs, PD-L1 granular staining was considered positive and the percentage of positive cells was scored.

### Statistical analysis

We used IBM SPSS Statistics for Windows, Version 24.0. (IBM Corp., Armonk, NY) for statistical calculations. The Spearman’s rank correlation test was used to assess the correlation in PD-L1 expression between primary lung and BM tumor cells and primary lung tumor and BM TILs.

## RESULTS

A total of 24 NSCLC patients were included in the study. The majority of patients were male (n = 20, 83.3%) and 16.6% were female (n = 4). Thirteen patients were at the age of 65 or higher, while 11 were younger than 65. We analyzed 48 paired primary lung tumors and BMs from 24 cases. There were 18 adenocarcinomas, 2 squamous cell carcinomas, 1 adenosquamous carcinoma, 1 pleomorphic carcinoma, 1 large cell carcinoma, and 1 combined large cell neuroendocrine carcinoma. The majority of our specimens were resection materials (n = 40, 83%; lung = 16, brain = 24), but biopsies were also included (n = 8, 17%; lung = 8). Lung biopsy or resection was performed before BM resection in 15 cases.

**FIGURE 1:**
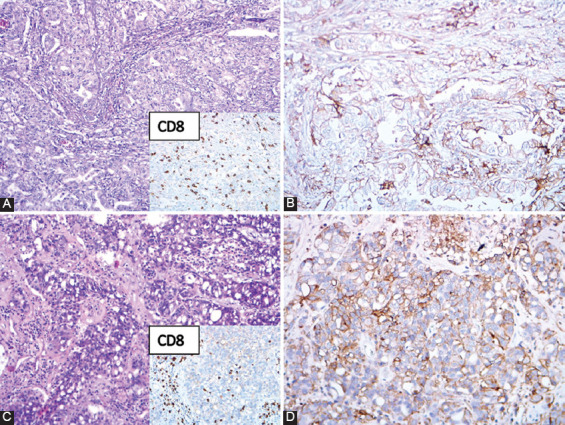
Concordant PD-L1 expression in primary lung tumor (A - HE, B - PD-L1 IHC) and brain metastasis (C - HE, D - PD-L1 IHC). CD8^+^ TILs are shown in the lower right corner of panels A and C. HE: Hematoxylin and eosin; IHC: Immunohistochemistry; PD-L1: Programmed death-ligand 1; TILs: Tumor-infiltrating lymphocytes.

**FIGURE 2:**
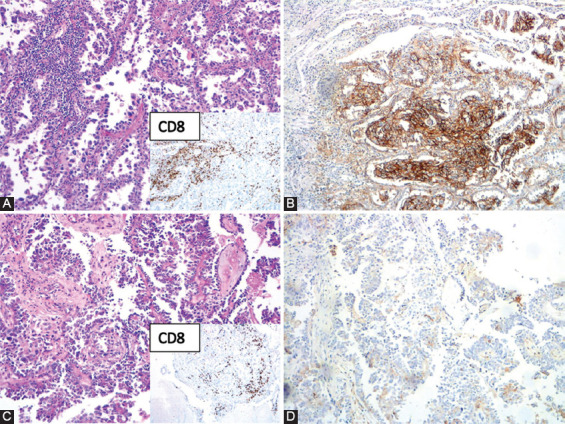
Discordant PD-L1 expression in primary lung tumor [80%] (A - HE, B - PD-L1 IHC) and brain metastasis [30%] (C - HE, D - PD-L1 IHC). CD8^+^ TILs are shown in the lower right corner of panels A and C. HE: Hematoxylin and eosin; IHC: Immunohistochemistry; PD-L1: Programmed death-ligand 1; TILs: Tumor-infiltrating lymphocytes.

**FIGURE 3:**
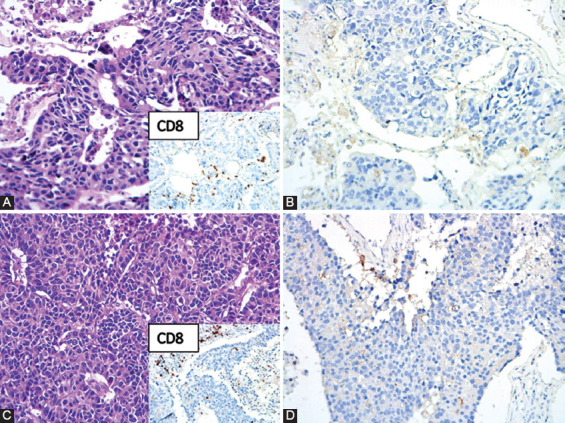
Concordant but weak PD-L1 expression in both primary lung tumor [1%] (A - HE, B - PD-L1 IHC) and brain metastasis [1%] (C - HE, D - PD-L1). CD8^+^ TILs are shown in the lower right corner of panels A and C. HE: Hematoxylin and eosin; IHC: Immunohistochemistry; PD-L1: Programmed death-ligand 1; TILs: Tumor-infiltrating lymphocytes.

**FIGURE 4:**
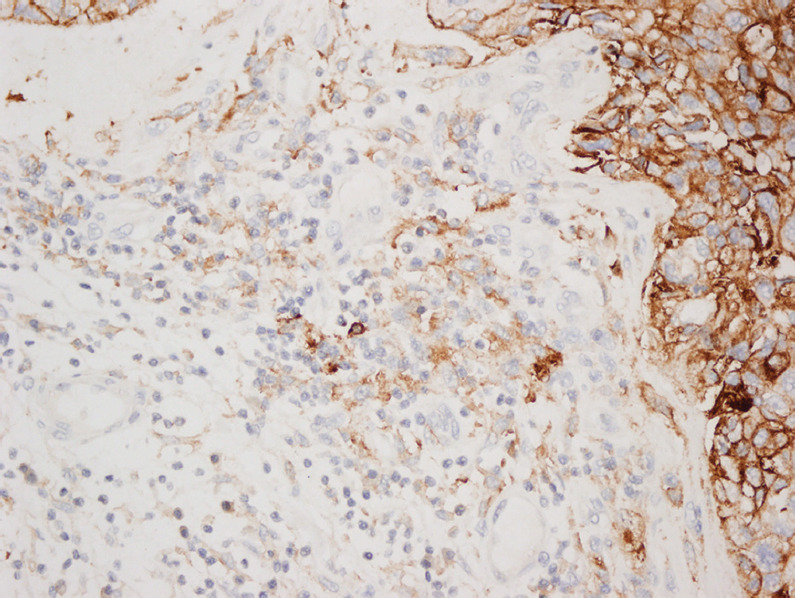
Programmed death-ligand 1 (PD-L1) immunoreactivity in tumor-infiltrating lymphocytes.

Out of 24 patients, 18 had PD-L1(+) tumor cells in primary lung tumor, while 6 had PD-L1(-); 17 had PD-L1(+) tumor cells in BM, while 7 had PD-L1(-). The results were exactly the same for PD-L1 expression on TILs in primary lung tumors and BMs (Table 1). Figures 1-4 show representative examples of PD-L1 expression between primary lung tumors and BMs.

**TABLE 1:**
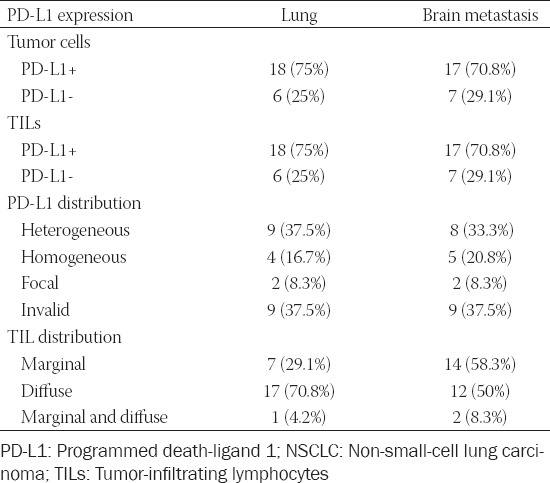
PD-L1 expression in 24 NSCLC patients

In primary lung tumors, PD-L1 positivity on tumor cells was observed in 25%, 37.5%, and 37.5% cases for the cutoff values <1%, 1–49%, and ≥50%, respectively (Table 2).

**TABLE 2:**
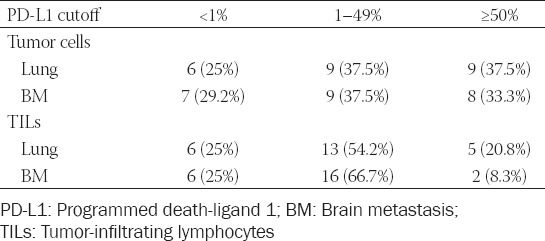
PD-L1 score in primary lung tumors and BMs

Among 18 cases of PD-L1(+) tumor cells in primary lung tumor, 9 patients were in both poor and strong positive groups. The results were almost the same for PD-L1(+) tumor cells in BMs, with 9 cases in poor positive group and 8 in strong positive.

The number of cases with negative PD-L1 expression on TILs was the same in primary lung tumors and BMs (n = 6). The number of cases in poor positive group was considerably higher compared to strong positive group in both primary lung tumors and BMs (in poor positive groups: 13 and 16 patients in primary lung tumors and BMs, respectively; in strong positive group: 5 and 2 patients in primary lung tumors and BMs, respectively) (Table 2).

The correlation of PD-L1 expression between primary lung and BM tumor cells is shown in Table 3. Fifteen patients (62.5%) had PD-L1(+) tumor cells in both primary lung tumor and BM. Three patients had PD-L1(+) tumor cells in primary lung tumor and PD-L1(-) tumor cells in BM (12.5%). Of particular interest were 2 patients who had PD-L1(-) tumor cells in their primary lung tumor but PD-L1(+) tumor cells in BM. Four patients had PD-L1(-) tumor cells in both their primary lung tumor and BM. PD-L1 expression on tumor cells was strongly correlated between the paired primary lung and BM tissues, in all cutoff groups.

**TABLE 3:**
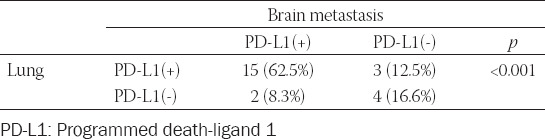
Correlation of PD-L1 expression in tumor cells

The correlation of PD-L1 expression between primary lung and BM TILs is shown in Table 4. The results are similar to the above results of PD-L1 expression on tumor cells between primary lung tumors and BMs.

**TABLE 4:**
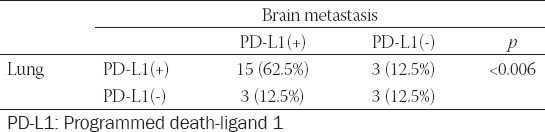
Correlation of PD-L1 expression in tumor-infiltrating lymphocytes

**FIGURE 5:**
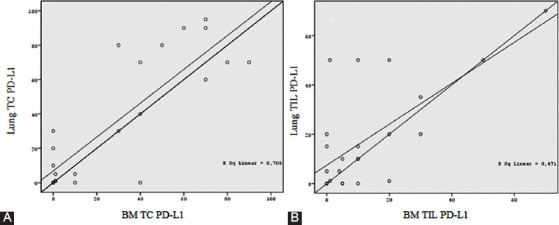
Correlation of PD-L1 expression between primary lung and BM TCs (A) and primary lung tumor and BM TILs (B). PD-L1 expression on TCs showed a stronger correlation between paired primary lung tumors and BMs compared to PD-L1 expression on TILs. TCs: Tumor cells; PD-L1: Programmed death-ligand 1; BM: Brain metastasis; TILs: Tumor-infiltrating lymphocytes.

As shown in Figure 5, PD-L1 expression on tumor cells showed a stronger correlation between the paired primary lung tumors and BMs compared to PD-L1 expression on TILs.

PD-L1 expression on tumor cells and TILs was evaluated in relation to CD3^+^/CD8^+^ TIL proportions and the intensity of CD8^+^ TILs between the paired primary lung and BM tissues.

**FIGURE 6:**
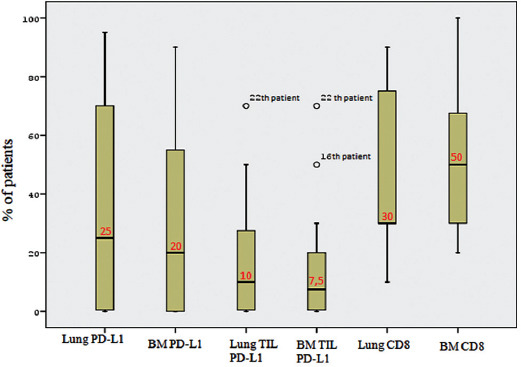
Box-plots of PD-L1 expression on tumor cells and TILs and CD8 expression between primary lung tumors and BMs. The median values are labeled with a red color. The results for PD-L1 expression on tumor cells and TILs were almost parallel (as can be seen from the average lines plotted towards the middle sections of the boxes and from the box sizes), while CD8^+^ TILs varied between primary lung tumors and BMs. PD-L1: Programmed death-ligand 1; BM: Brain metastasis; TILs: Tumor-infiltrating lymphocytes.

Figure 6 displays the box plots of PD-L1 expression on tumor cells and TILs and CD8 positivity in primary lung tumors and BMs. The results for PD-L1 expression on tumor cells and TILs were almost parallel (as can be seen from the average lines plotted towards the middle sections of the boxes and from the box sizes), while CD8^+^ TILs varied between primary lung tumors and BMs. The intensity of CD8+ TILs was concordant in only 54.16% of the paired primary lung tumors and BMs.

## DISCUSSION

In our study, PD-L1 expression on tumor cells was statistically comparable between primary lung tumors and BMs, indicating that the concordance of PD-L1 expression on neoplastic cells between primary NSCLCs and their synchronous BMs is high. This means that the evaluation of PD-L1 in either primary lung tumor or BM can be considered valid in the selection of anti-PD-1/PD-L1 immunotherapy for patients.

PD-L1 expression on TILs was also similar in ratios between primary lung tumors and BMs in our study. This finding is interesting because the microenvironment of the brain is quite different from that of the lung and PD-L1 expression on TILs does not seem to be influenced by those differences. On the other hand, when we compared the intensity of CD8^+^ TILs between primary lung tumors and BMs, only 54.16% (13/24) of the tumor pairs were concordant. Due to this low concordance of CD8 intensity in TILs, CD8^+^ TILs may not be applicable as a therapeutic target in primary lung tumors and BMs.

Mansfield et al. investigated temporal and spatial discordance of PD-L1 expression and TILs between paired primary lesions and BMs in 73 lung cancer cases and showed different results from ours [9]. They reported significant changes in the classifications as many BMs lacked PD-L1 expression, TILs, or both, even when they were present in the primary lung cancer samples. [9]. The fact that Mansfield et al. also considered the spatial and temporal dimensions of PD-L1 expression in lung cancer does not seem to be the cause of the difference in the results between their and our study. The discrepancy, however, might be due to a difference in the race of patients, the fact that they used biopsy specimens rather than complete resection specimens, or because more time passed between the initial diagnosis of lung tumor and BM in some of their patients (i.e., they had different patient groups). It is also possible that the results vary between their and our study due to different cutoff points or PD-L1 antibodies used. Mansfield et al. used the PD-L1 clone E1L3N and considered lesions with 5% or greater PD-L1 expression as positive [9].

A similar study was conducted by Berghoff et al. who investigated PD-L1 expression and TILs in BMs of small cell lung cancer [10]. An interesting point when comparing our and their results is that while they reported membranous PD-L1 expression on tumor cells in 75% of BMs, they showed that only 25% of TILs in BMs were PD-L1 positive. In another study, Berghoff et al. reported a clear topographical and statistical correlation of TIL infiltration with PD-L1 expression in patients with melanoma and BMs [18]. While not directly comparable, the findings of those two studies are in line with ours.

Our study showed that NSCLC BMs harbor an active immune microenvironment with the presence of TILs and PD-L1 expression.

The small number of cases as well as the unequal distribution of histological subtypes of NSCLC are limitations of our study.

Further research on this subject is required to gain a deeper insight into the mechanisms underlying the presence CD8^+^ TILs and PD-L1 expression in NSCLC.
